# Data Completeness and Acceptability of Annual Feedback Reports and Tuberculosis Surveillance in Spain, 2018-2024: Mixed Methods Analysis

**DOI:** 10.2196/93719

**Published:** 2026-09-16

**Authors:** Allegra Chatterjee, Alvaro Roy, Rocio Amillategui, María Sastre, Christine Schwarz, Rosa Cano, Laura Herrera-León, Zaida Herrador, Spanish Working Group on Tuberculosis

**Affiliations:** 1 European Centre for Disease Prevention and Control, Fellowship Programme/EPIET, Stockholm, Sweden; 2 Centro Nacional de Epidemiología, Instituto de Salud Carlos III, calle Sinesio Delgado nº6, Pabellon 12, Madrid, Madrid, 28029, Spain, 34 91 8222012; 3 Network Biomedical Research Centre in Epidemiology and Public Health (CIBERESP), Madrid, Spain; 4 Health Division for HIV, STI, Viral Hepatitis and Tuberculosis Control, Ministry of Health, Madrid, Madrid, Spain; 5 Centro Nacional de Microbiologia, Instituto de Salud Carlos III, Majadahonda, Madrid, Spain; 6 See Acknowledgments

**Keywords:** tuberculosis, surveillance, Spain, public health, infectious diseases

## Abstract

**Background:**

Surveillance of tuberculosis (TB) is challenging given the typically long course of the disease and treatment as well as the often complex socioeconomic factors that are associated with it. In Spain, surveillance is decentralized across autonomous communities (CCAAs), leading to additional challenges in heterogeneity and data quality.

**Objective:**

We aimed to quantify changes in internal data completeness following introduction of annual regional surveillance quality reports and assess the acceptability and perceived usefulness among CCAAs.

**Methods:**

This mixed methods assessment consisted of 2 stages. First, we quantitatively assessed the internal completeness of 40 key TB variables for all reported cases from 2018 to 2023, comparing 2 periods: before (2018‐2020) and after (2021‐2023) the implementation of feedback reports. Mean completeness for each variable was calculated for each period, and the differences were compared using either the paired *t* test or Wilcoxon signed rank test depending on whether differences were normally distributed. Analyses were conducted nationally and by CCAA as well as for different variable groups (patient-, illness-, and laboratory-related groups). For the second stage, we circulated the results to regional TB surveillance focal points, along with a survey to assess acceptability, perceived usefulness of reports, and barriers to data completeness.

**Results:**

There were 25,229 reported cases across the study period: 13,328 in period 1 (2018‐2020) and 11,901 in period 2 (2021‐2023). Nationally, mean completeness increased from 66% in period 1 to 76.2% in period 2 (+10.2%, *P*<.001). Improvements were greatest for laboratory-related (+13.9%, *P*=.001) and illness-related variables (+12.8%, *P*<.001), while patient-related variables showed minimal change that was not statistically significant (+2.4%, *P*=.48). Most CCAAs (16/19, 84%) demonstrated improved completeness in period 2, though substantial regional variation persisted. Of the 19 CCAAs, 18 responded to the survey. Respondents reported high acceptability of the surveillance system and considered feedback reports useful. Challenges included resource constraints, system interoperability between laboratory and surveillance, and patient follow-up.

**Conclusions:**

Following the introduction of annual feedback reports, which were well accepted by all regional stakeholders, Spain saw significant improvements in TB data completeness. Sustained feedback mechanisms, streamlined reporting requirements, and improved integration between epidemiological and laboratory systems are key to strengthening TB surveillance.

## Introduction

Tuberculosis (TB) remains the world’s deadliest infectious disease, despite being both preventable and usually curable [1]. In 2024, an estimated 10.7 million people worldwide developed TB, corresponding to an incidence rate of 131 cases per 100,000 population [2]. The same year, 11.5% of those diagnosed with TB died from the disease, a total of 1.2 million people.

To address the global TB burden, in 2014, the World Health Organization (WHO) launched the End TB Strategy, aiming to reduce incidence by 90% and deaths by 95% by 2035, compared with 2015 levels [3]. Although there is large regional variation, the number of TB cases and deaths globally decreased in 2024, following 3 consecutive years of increases due to disruptions caused by the COVID-19 pandemic [2]. The WHO European Region has achieved substantial progress since WHO targets were introduced, with a 39% reduction in incidence rate observed by 2024 [2]. However, significant challenges remain in the region; according to a 2026 report, multidrug-resistant TB rates are 7 times higher in Europe than the global average, 21% of TB cases are undiagnosed or unreported, and 22% of people who start treatment are not followed up after 1 year [4].

As countries continue to strive to meet these goals, robust TB surveillance systems are essential, enabling monitoring of trends in incidence and mortality, detecting outbreaks, identifying affected populations to create targeted interventions, and evaluating the impact of TB control measures to ensure accountability for national and international commitments. However, achieving comprehensive and accurate TB surveillance remains challenging. Common barriers identified in European countries include fragmented integration between clinical and laboratory information systems and heterogeneity in reporting across jurisdictions [5], as well as loss to follow-up during the long treatment courses required, particularly for vulnerable populations such as migrants or people experiencing homelessness [6]. Regular assessments of TB surveillance systems are therefore critical to ensure their effectiveness, identify gaps, and prioritize improvements.

In Spain, reported TB cases and hospitalizations showed a gradually declining trend between 2012 to 2020 [7]; however, recent surveillance reports show a slight rebound, with the notification rate rising from 7.7 to 8.8 per 100,000 population from 2020 to 2024 [8]. This trend was observed among children as well as adults in Spain; although a lower incidence is generally observed among children, following a decrease after 2012, increases have been observed in both notification and hospitalization rates among this group since 2021 [9]. Nevertheless, compared with global figures, the incidence and deaths rates are low, with WHO recategorizing the country from incidence group “low moderate” in 2015 to “low” by 2025 [2].

However, the decentralized nature of surveillance presents a challenge, with each of the 17 autonomous communities (CCAAs) and 2 autonomous cities (Ceuta and Melilla) having their own surveillance systems, with annual integration of data at the national level. In 2021, a rapid internal review by the National Center for Epidemiology (CNE) recognized that there were deficiencies in the completeness of some variables and a large degree of variation among CCAAs. To support improvements in TB surveillance data quality, the CNE began sharing annual personalized feedback reports to each CCAA applying to submissions of consolidated annual data from the year 2021 onward. These reports summarize completeness across TB surveillance variables, highlighting any inconsistencies identified and identifying areas for improvement.

We performed a mixed methods study aiming to quantify changes in internal data completeness following the introduction of annual regional feedback reports on the quality of surveillance data and to assess acceptability and perceived usefulness among CCAAs. The purpose of this was to evaluate whether this quality improvement initiative of annual feedback reports should be recommended to continue and, if so, whether they should be refined. The study comprised 4 objectives: (1) quantitatively evaluate the internal completeness of TB surveillance data before and after implementation of feedback reports, (2) qualitatively assess the acceptability and perceived usefulness among CCAAs of both the current surveillance system and the feedback reports, (3) update annual feedback reports based on findings from objectives 1 and 3, and (4) assess the impact of the newly updated reports on completeness of TB surveillance data for 2024.

## Methods

### Data Sources

**Figure 1.**
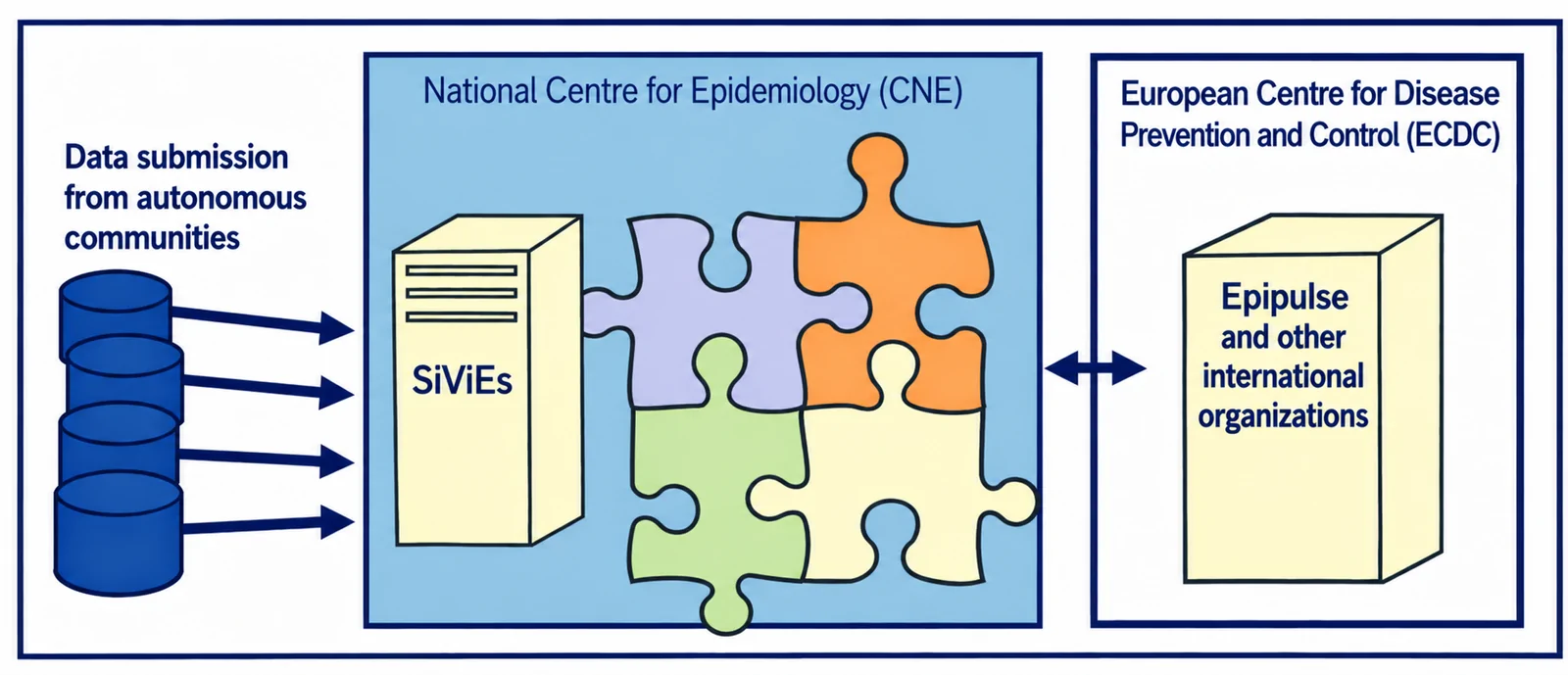
Flow of data from autonomous communities into the national Spanish Surveillance System (SiViEs Plus) and the ECDC.

The Spanish TB national surveillance system is passive, case-based, and at the population level; each CCAA is responsible for collecting and annually reporting patient-level data on predefined variables into the Spanish Surveillance System (SiViEs Plus) according to the TB protocol published by The National Epidemiological Surveillance Network (La Red Española de Vigilancia Epidemiológica [RENAVE]; Figure 1) [10].

We extracted data on 40 key variables from all reported cases in Spain from the SiViEs Plus database between January 1, 2018, and December 31, 2023. We divided variables into 3 categories: (1) patient-related information (11 variables), (2) illness-related information (18 variables), and (3) laboratory-related information (11 variables).

For the second aim, we developed a questionnaire based on a literature review and consulting with TB and surveillance experts at the CNE. The 17-question online questionnaire included both closed and open-ended questions to gather information on the perceived usefulness of the annual feedback reports, challenges around TB surveillance, and suggestions on how to improve TB surveillance in Spain. We piloted the questionnaire with the TB surveillance lead from 1 CCAA. In April 2025 and May 2025, we circulated the final version via Google Forms among the Spanish TB Surveillance Working Group. The survey was completed by the lead individual responsible for TB surveillance in each CCAA; these individuals were considered best placed to respond on behalf of their CCAA as they have an overview of all areas of TB surveillance in their region, they are responsible for submission of data at the national level, and they are the person who receives the annual feedback reports. The survey invitation emphasized that results would be reported anonymously and not attributable to any CCAA or individual.

### Ethical Considerations

This study used anonymized data from RENAVE in Spain. RENAVE collects data on cases notified through the national reporting electronic platform SiViEs Plus, and it is hosted by the CNE. These data are generated under a legal public health mandate and are routinely used for epidemiological monitoring. As the study involved secondary analysis of anonymized administrative surveillance data accessed under legally mandated confidentiality safeguards, formal ethics committee approval was not required. This exemption is consistent with Spanish and European data protection regulations governing the processing of anonymized public health surveillance data, including Ley Orgánica 3/2018 de Protección de Datos Personales y Garantía de los Derechos Digitales (LOPDGDD) and Reglamento (UE) 2016/679 (GDPR).

Survey data were submitted by a regional focal point from CCAAs in Spain. For survey participation, informed consent was obtained from participants prior to beginning the survey. Participants were made aware that taking part was voluntary, that there would be no consequences for not taking part, and that they could withdraw from participating at any time without consequence. One response was obtained from each CCAA; however, responses were reported anonymously in the analysis and write-up to maintain confidentiality. All data were stored and analyzed within secure, password-protected ISCIII systems in compliance with institutional data protection policies.

### Definitions

Internal completeness and acceptability surveillance attributes were chosen as they align with those set out by the European Center for Disease Control and Prevention (ECDC) technical handbook “Data quality monitoring and surveillance system evaluation” [11] as key for high-functioning surveillance systems.

We defined internal completeness according to ECDC [11] as “the number of completed data fields out of the total number of data fields (unknown and missing items should be included in the denominator).” The denominator was not always equal to the total number of cases; for example, the variable “number of years living in Spain” was only applicable to cases born outside of Spain.

### Surveillance Cycle and Feedback Reports

TB data are submitted continuously by CCAAs to SiViEs Plus; however, each annual dataset is only considered complete once regions confirm that all records for the previous year have been uploaded, typically in Q2 of the following year (eg, annual data for 2021 would be consolidated in Q2 of 2022).

Since 2022, following the annual submissions from the CCAAs, the national level (CNE) performs data validation and generates feedback reports highlighting missing or inconsistent information (see Multimedia Appendix 1 for an example report in the original Spanish and in an English translation that was carried out using the generative artificial intelligence [AI] tool, Copilot). These reports are typically shared with CCAAs between May and July, with variation in timing depending on when each region finalizes its initial submission. CCAAs subsequently update their annual data before final national consolidation, which usually occurs in the third quarter (Q3). The feedback reports disseminated in a given year are therefore designed to inform and improve data reporting practices for the year prior. As feedback reports began being disseminated in 2022, the year 2021 was considered the first year in which reporting could be influenced by the feedback process.

### Data Analysis

#### Part 1: Internal Completeness Analysis

We first calculated the percentage internal completeness for each of the 40 variables across each year from 2018 to 2023. For each variable, we then calculated the mean percentage completeness (see the definition in the “Definitions” section) for two 3-year periods: (1) before reports were implemented (period 1): 2018‐2020 and (2) after reports were implemented (period 2): 2021‐2023.

For each period, we then calculated a mean across all variables by summing the mean percentage completeness for each variable and dividing by 40; we used the same approach to calculate means for period 1 and period 2 for each of the 3 subcategories (patient-, illness- and laboratory-related variables).

For each variable, we calculated the difference in completeness before and after implementation of feedback reports by subtracting the mean percentage completeness of that variable in period 1 from that of period 2.

For all variables combined and for each of the 3 subcategories, we used the Shapiro-Wilk test to examine whether the differences in completeness between the 2 periods were normally distributed or not; if found to be normally distributed, the paired *t* test was used to test for statistical significance (set at 5%). Otherwise, the Wilcoxon signed-rank test was used. This analysis was carried out at the national level and separately for each CCAA. All analyses were performed using R version 4.4.1.

We also carried out a sensitivity analysis to examine whether the COVID-19 pandemic may have impacted the results. We repeated the internal completeness analysis after removing the 2 years of 2020 and 2021; therefore, the comparison periods each contained 2 years (2018‐2019 and 2022‐2023), and we compared the results with those obtained for the original comparison periods.

#### Part 2: Survey Analysis

Together with the questionnaire, we circulated to each CCAA a preliminary analysis of national-level data compared with the regional data. This included a written summary and figures showing changes in completeness of surveillance data with respect to the 3 categories of variables across the 2 periods, comparing the values for their CCAA with the national averages (see Multimedia Appendix 1).

We calculated the proportion of respondents in each category of closed-ended questions. We reviewed free-text responses and grouped them into categories based on recurring content and meaning.

#### Part 3: Annual Report Updates and Continuous Quality Improvement

In June 2025, to support the CCAAs to make continual improvements in TB surveillance, we shared with the regions a summary of the qualitative analysis, along with a description of changes made to the upcoming feedback report (for 2024 data submission) based on the findings. Following this, we later performed an analysis of the 2024 TB surveillance data to determine whether the updated feedback report supported further improvements, as part of a continuous improvement cycle.

## Results

### Part 1: Internal Completeness Analysis

TB surveillance data for 25,229 reported cases were extracted across the study period. In period 1 (2018‐2020), there was a total of 13,328 cases, and in period 2 (2021‐2023), there was a total of 11,901 cases. Comparing internal completeness of 40 variables across period 1 and period 2, there was a mean improvement of 10.2% (Wilcoxon signed rank test *P*<.001), from 66% in period 1 to 76.2% in period 2, with 78% (31/40) variables showing an increase (Table 1). No change was observed for 4 (10%) of the 40 variables (age, sex, TB classification, date of case report), which all had a completeness rates ≥99.9% in period 1, while 5 (13%) of the 40 variables had a decrease in completeness (country, CCAA, province and municipality of residence, and mother number of years in Spain; Table 1).

Patient-related variables had the highest overall completeness for both periods (80.3% for period 1 and 82.7% for period 2). However, the mean increases of 2.4% across the 11 variables was lower than that of other variable groups and was not statistically significant (Wilcoxon signed rank test *P*=.48). All 5 variables with a decrease in completeness were within this category.

Illness-related variables significantly increased by 12.8% (paired *t* test: t_17_=–5.185, *P*<.001), from 64.6% in period 1 to 77.4% in period 2. Of the 18 variables within this group, 2 did not increase (TB classification and date of case report) as they were already at 100% completion during period 1.

The greatest improvement was seen with laboratory-related variables, with an increase of 13.9% (paired *t* test: t_10_=*–*4.392, *P*=.001) from 53.9% in period 1 to 67.7% in period 2. All 11 variables increased, the largest of which was for “date of laboratory diagnosis,” which increased by 37.5%.

**Table 1.** Completeness of tuberculosis (TB) surveillance variables at the national level comparing period 1 (2018‐2020) with period 2 (2021‐2023) in Spain.

Variable	Mean completeness period 1, %	Mean completeness period 2, %	Difference (period 2 – period 1), %	*P* value for the difference
Patient variables				
Date of birth	85.8	98.6	+12.8	N/A^a^
Age	99.9	99.9	0	N/A
Sex	100	100	0	N/A
Country of residence	99.6	98.2	−1.4	N/A
Autonomous community of residence	99.5	98	−1.5	N/A
Province of residence	99.4	97.2	−2.2	N/A
Municipality of residence	99.1	97.6	−1.5	N/A
Country of birth	87.4	90.8	+3.4	N/A
Number of years in Spain	47.3	62.3	+15.0	N/A
Mother country of birth	7.6	12.3	+4.6	N/A
Mother number of years in Spain	57.3	55.3	−2.0	N/A
Mean of all patient variables	80.3	82.7	+2.4	.48
Illness variables				
TB classification	100	100	0	N/A
Date of case report	100	100	0	N/A
Date of symptom onset	79.4	84.9	+5.5	N/A
Country of case report	88.9	89.5	+0.6	N/A
Autonomous community of case report	70.1	71.9	+1.8	N/A
Province of case report	35.2	66.1	+30.9	N/A
Municipality of case report	32.6	64.5	+31.9	N/A
Importation	85	92.4	+7.4	N/A
TB location	97.4	98	+0.7	N/A
Previous treatment	64.9	82.1	+17.2	N/A
Directly observed treatment	64.9	82.1	+17.2	N/A
Associated with outbreak	27.7	54	+26.3	N/A
Date of treatment initiation	70.8	85.1	+14.4	N/A
Date of treatment end	44.8	57.1	+12.3	N/A
Treatment result	63	77.8	+14.8	N/A
Follow-up result	15.4	39.3	+23.8	N/A
Hospitalization	76.9	90.8	+13.9	N/A
Death	63.6	79	+15.4	N/A
Mean of all illness variables	64.6	77.4	+12.8	<.001
Laboratory variables				
Date of lab diagnosis	41.7	79.2	+37.5	N/A
Causal agent	73.3	76.6	+3.4	N/A
Lab test carried out	35.2	50.6	+15.4	N/A
Lab result: isolation	93.3	93.6	+0.3	N/A
Lab result: visualization	92.9	93.0	+0.1	N/A
Lab result: antimicrobial susceptibility	47.8	66.2	+18.4	N/A
Etambutol resistance test	31.9	50.2	+18.3	N/A
Isoniazide resistance test	35.4	49.9	+14.5	N/A
Pirazinamide resistance test	33.6	46.8	+13.2	N/A
Rifampicine resistance test	35.8	50.4	+14.6	N/A
HIV test	71.7	88.7	+17.1	N/A
Mean of all laboratory variables	53.9	67.7	+13.9	.001
All variables	66	76.2	+10.2	<.001

a N/A: not applicable.

**Figure 2.**
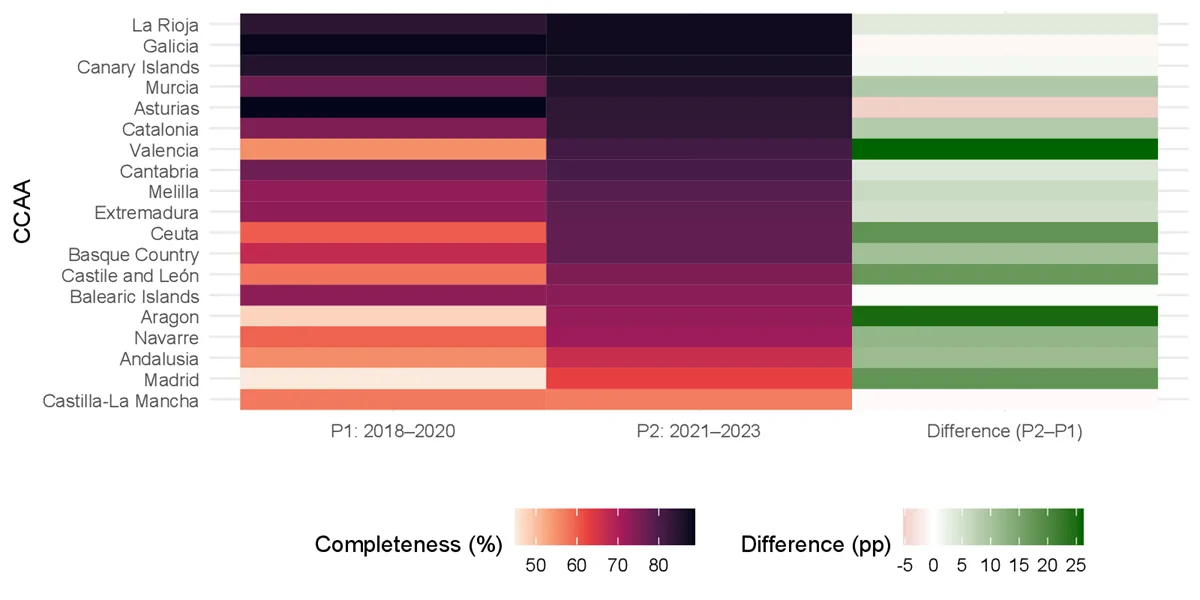
Heat map showing mean percentage completeness of 40 tuberculosis (TB) variables reported by autonomous communities (CCAAs) in period 1 (P1: 2018‐2020) and period 2 (P2: 2021‐2023) in Spain.

There was a large degree of variation across the 17 CCAAs and the 2 autonomous cities (Figure 2). For period 1, mean annual percentage completeness across all variables ranged from 44.9% to 88.7%, and for period 2, it ranged between 56.9% and 87.5%. Comparing period 1 with period 2, differences in completeness ranged from a decrease of 5.2% to an increase of 26.2% in period 2. For all regions, 84% (16/19) had an improvement in period 2, 69% (11/16) of which had statistically significant improvements (*P*<.05). Of the 3 (3/19, 16%) CCAAs where a decrease in completeness was observed, only 1 was statistically significant.

Results obtained were similar across all analyses when the pandemic years of 2020 and 2021 were excluded (Table S2 in Multimedia Appendix 2). Across all 40 variables, there was a statistically significant increase of 12.5% (*P*<.001), compared with 10.2% (*P*<.001) in the original analysis. For patient variables, results were identical to the original analysis, with a nonstatistically significant increase of 2.4% observed. Differences between the 2 periods were slightly greater for illness-related variables and laboratory-related variables when excluding the pandemic years: 14.8% (*P*<.001) compared with 12.8% (*P*<.001) and 18.9% (*P*<.001) compared with 13.9% (*P*=.001), respectively.

### Part 2: Survey Analysis

#### Satisfaction With Surveillance System

Overall, 95% (18/19) of CCAAs completed the online questionnaire. All 18 respondents reported that having high-quality TB surveillance data at the national level is either “very important” (17/18, 94%) or “important” (1/18, 6%). However, satisfaction with the current system varied; although most reported feeling either “very satisfied” (3/18, 17%) or “satisfied” (8/18, 44%), 33% (6/18) were neutral, and 1 (1/18, 6%) CCAA reported feeling unsatisfied. Regarding the completeness of data reported from the CCAAs, results were similar, with most reporting being either very satisfied (2/18, 11%) or satisfied (9/18, 50%), while 33% (6/18) were neutral and 1 respondent was unsatisfied. Satisfaction with the current system did not appear to vary depending on the completeness of data submitted by CCAAs.

Reported issues included the challenging context of TB surveillance given the complex interactions between the social, cultural, and health care–related aspects of the disease. Some respondents felt that the amount of information requested for each case was too high and that some variables may not be necessary, particularly those that are hard to define or calculate such as time to follow-up.

Respondents suggested that the current system could be streamlined to focus on high-priority indicators for TB control. Several respondents called for clearer guidance on variable definitions and their categories, as well as integration of case follow-up information given the long disease course and, in particular, how to manage surveillance and declaration of cases that transfer between CCAAs.

In addition, the need for a strong system for identifying unreported cases and the extent of under-ascertainment in Spain was highlighted; suggestions included examining differences in proportions (eg, confirmed or extrapulmonary TB) across regions to detect inconsistencies that could indicate under-ascertainment.

#### Usefulness of Annual Feedback Reports

The annual feedback reports were considered to be useful by all respondents; 17% (3/18) felt they are “extremely useful,” 61% (11/18) “very useful,” and 22% (4/18) “moderately useful.” . As with the attribute of usefulness, this did not seem to be associated with how well the region performed with respect to data completeness. Free-text responses indicated that CCAAs felt that reports help improve data quality by identifying missing or inconsistent entries. Some respondents noted that reports contribute to an environment of continuous quality improvement, stimulating internal reviews and priority areas to improve regional surveillance, as well as helping to evaluate interventions. Several also highlighted their value in raising awareness among TB units, nurses, IT staff, and public health leadership.

Of respondents, 78% (14/18) and 83% (15/18) felt that the format and content of the feedback reports, respectively, were appropriate, while 22% (4/18) and 17% (3/18) felt that the format and content of the feedback reports, respectively, were partially appropriate. None of the participants reported that they were not appropriate. Suggestions for improvements included highlighting the poorest performing variables within each section, considering simplifying data, and including fewer variables so that those of higher importance can be prioritized. In addition, one respondent suggested that coherence and internal validity between variables could be highlighted as well as completeness. Of all respondents, 72% (13/18) thought that the timing of report distribution allowed for improvements before the next data submission cycle; however, 22% (4/18) felt that they were sent too late, and 1 respondent thought they arrived too early. One person commented that they would prefer to receive reports more frequently, every 4 months. In terms of dissemination, 50% (9/18) of respondents reported sharing the reports within their regional teams, including with epidemiologists, TB units, IT teams, primary care, hospital services, and regional public health directors and leadership. Better-performing CCAAs appear to be more likely to circulate reports to colleagues; among the 5 regions with highest period 2 completeness, 4 reported sharing reports with colleagues; however, none of the bottom 5 performing regions reported sharing the reports.

#### Barriers and Enablers

Barriers to complete TB data reporting varied across regions but commonly included limited human resources and time (15/18, 83%); technical restrictions (9/18, 50%); insufficient training (5/18, 28%), which is exacerbated by high staff turnover; and lack of clear surveillance guidelines (4/18, 22%). Many CCAAs had already implemented measures to improve surveillance, including integration of laboratory and microbiological data into their surveillance systems, local data validation prior to submission, feedback loops between regional TB units and central teams, automated quality checks to flag inconsistencies within information systems, and continuous improvements to electronic reporting platforms.

Suggestions for broader system improvements included online training resources for surveillance staff and continuous quality improvement initiatives, including maintaining the annual feedback reports and possibly increasing their frequency, improved variable definitions in the national protocol to ensure consistency, and national guidelines for follow up of cases who move between regions.

### Part 3: Annual Report Updates and Continuous Quality Improvement

In June 2025, to support CCAAs with making continuous improvements in TB surveillance, we shared with regions a summary of Part 1 and Part 2 of the assessment, along with a description of changes made to the upcoming feedback reports (for 2024 data submission) based on the findings:

- Number of variables included reduced to the 40 key variables included in the analysis
- Variables separated into 3 groups to coincide with the analysis: patient, illness, laboratory
- Addition of various cross-checks, including infective agent classification and diagnostic test used, date of case registration and result of treatment, date of treatment completion, and result of treatment; also, the addition of several new cross-checks related to antimicrobial resistance testing
- Time to diagnostic and time to treatment added to reports as a new variable, calculated using the difference between the date of diagnosis and date of symptom onset and between the date of initiation of treatment and date of symptom onset, respectively

Following this, we analyzed the preliminary 2024 TB surveillance data to determine whether the updated feedback report supported further improvements, as part of a continuous improvement cycle. We found that, at the national level, mean completeness across all variables increased to 78% in 2024 (compared with 66% in 2018‐2020 and 76% in 2021‐2023).

## Discussion

### Summary of Principal Findings

This study aimed to quantitively assess the internal completeness of TB surveillance data before and after implementation of feedback reports in Spain and to qualitatively assess the perceived usefulness among CCAAs of both the current surveillance system and the feedback reports. We identified significant improvements in completeness across most variables and regions following the introduction of annual feedback reports, although there was some variation across both type of variable and CCAAs. At the national level, mean completeness increased from 66% in 2018‐2020 to 76.2% in 2021‐2023 (with nearly four-fifths of variables indicating improvements) and increased further to 77.8% in 2024. Both the surveillance system and feedback reports were generally viewed as acceptable and useful among regional stakeholders included in the survey. The additional study aiming to update the feedback reports for the 2024 surveillance cycle based on our findings and assess the potential impact of the updated reports on TB surveillance data completeness found that mean completeness continued to improve in 2024 relative to previous years.

### Principal Findings in Context

Although the highest completeness levels were seen among patient-related variables, no statistically significant improvement was observed in this group. This is partly due to many variables being at or close to 100% completeness in period 1, leaving minimal opportunity for further improvement. This is in line with findings from other European Union (EU) and European Economic Area (EEA) countries, where, in 2023, most countries had complete or near complete data (>95%) for variables requiring mandatory reporting to the ECDC, such as age and sex [12]. Patient-related variables that continued to have low completeness rates in period 2 were those that applied to individuals born outside of Spain: number of years in Spain, mother country of birth, and mother number of years in Spain (62.3%, 12.3%, and 55.3% period 2 completeness, respectively). This is pertinent, as individuals from countries of origin with higher TB prevalences may have an increased risk of importation; therefore, efforts should be made to improve data collection for these variables.

Although illness-related variables had similar percentage completeness (77.4%) to that of patient-related variables in period 2, a larger improvement of 12.8% in illness-related variables was observed relative to period 1. However, some variables remained poorly completed, including follow-up result, which, despite a 23.8% increase, had 39.3% completeness in period 2. Supporting this, survey respondents called for improved protocols and better integration of case follow-up and laboratory information, particularly for patients who move between CCAAs. The respondents’ call is in the context of the WHO recommendations for a 6-month regimen of 4 first-line drugs (and longer treatment and additional drugs for resistant TB) [13], which complicates patient follow-up. Continuity of TB treatment is essential for effective control of the disease, particularly with increasing movement of individuals both within and between countries across Europe [14]. In particular, interruption of treatment among mobile populations (including migrants, refugees, and asylum seekers) is a recognized challenge in Europe, often leading to poorer health outcomes, higher risk of transmission, and the emergence of resistant strains [15]. In line with this study, evidence from other countries has found that variability regarding migrant health policies and unclear or absent referral mechanisms can hinder treatment continuity [6,14,16].

Improvements were most pronounced for laboratory-related variables, with a mean increase of 13.9%; however, increases were particularly pronounced for those with lower baseline completeness, such as for date of laboratory diagnosis and variables related to antimicrobial susceptibility testing. Although this highlights substantial improvements in capturing microbiological information, challenges persist due to the lack of interoperability between clinical and laboratory systems. Despite improvements, some laboratory variables continue to have low completeness in period 2, including lab tests carried out (50.6%) and results for the 4 included antimicrobial resistance testing variables, which ranged from 31.9% to 50.4%. This finding reinforces the need to consolidate mechanisms for the automatic integration of microbiological data into epidemiological surveillance, as demonstrated effectively in some CCAAs. In Catalonia, for example, which ranks higher than most CCAAs with respect to completeness in period 1 (7th) and period 2 (6th), laboratory results are linked to the regional surveillance system [17]. At the European level, models such as the MiBa (Danish Microbiology Database) in Denmark [18], Osiris system in the Netherlands [19], and MycobNet network in the United Kingdom [20] enable automated laboratory result reporting and the genomic integration of *Mycobacterium tuberculosis* strains, improving outbreak detection and resistance surveillance.

This finding that variables requiring integration between clinical and laboratory systems or ongoing follow-up were the most incomplete is consistent with the ECDC [11] and United States Centers for Disease Control and Prevention (CDC) guidance [21], emphasizing that data involving multiple sources or complex definitions are generally more vulnerable to missingness. In line with this, across EU/EEA countries in 2023, the availability of data related to clinical and laboratory information was lower than for patient-related variables, for example for previous treatment history (86%), treatment outcome at 12 months (61.5%), and HIV status (39.3%) [12].

At the regional level, 16 of Spain’s 19 CCAAs (84%) saw an improvement in period 2, although the magnitude varied widely, ranging from modest gains to more than 25% increases. The one region where a statistically significant decrease was observed already had a high level of completeness in period 1 (the 5th highest among all CCAAs). These disparities reflect the decentralized nature of the Spanish surveillance network RENAVE. This is in line with other countries with decentralized surveillance systems, where regional disparities have been found in evaluations of the surveillance systems, for example in Italy where northern regions were found to report acute viral hepatitis cases in a more timely manner than central and southern regions [22].

Survey findings provided important explanatory context. Respondents were generally satisfied with TB surveillance in Spain and considered it to be important, indicating high level of acceptability and perceived usefulness of the system, providing a strong foundation for future quality improvement initiatives. There appeared to be a possible association between higher-performing CCAAs and the extent to which the feedback reports are disseminated among colleagues within the region; further investigation would be required to understand whether this is because the reports are more effective at driving improvements if disseminated more widely or simply that higher-performing CCAAs have both better communication channels and better surveillance systems.

Overall, our findings show an improvement in internal completeness of TB surveillance data following implementation of feedback reports and positive perceptions of these reports among regional stakeholders. Although a causal link is difficult to establish, our findings suggest that systematic data quality monitoring and feedback may help to strength surveillance, consistent with international guidance that emphasizes regular evaluation as a cornerstone of high-performing systems [11,21]. The observed improvements, together with respondents’ endorsement of feedback reports, suggest that feedback cycles may contribute to improved completeness and system acceptability and continuous quality improvement.

### Limitations

This analysis is subject to several limitations. First, due to the descriptive nature of the analysis, it was difficult to establish a causal link between the observed improvements in data quality and the implementation of the feedback reports. For this reason, we included the qualitative element of the survey, but it is not possible to rule out other explanations for the observed results, such as improvements in surveillance across all areas or increased focus on infectious diseases after the COVID-19 pandemic. Furthermore, although improved completeness likely reflects better data capture, it does not guarantee data accuracy; although the feedback reports were designed to examine this by including validity cross-checks, there are variables for which this was not possible. Case ascertainment and the sensitivity of the system—the number of cases captured by the system divided by the true number of cases in the community [11]—were flagged as a potential area of risk by survey respondents and remains an important attribute of surveillance that has yet to be explored.

There were additional limitations related to the survey component. The survey was circulated to the lead individual responsible for TB surveillance in each CCAA, meaning that there was only one response per region. This individual is the person receiving the feedback reports so they were considered best placed to respond to our survey; however, it is possible that this could introduce bias and may not fully reflect the breadth of opinions of individuals in the CCAA. In addition, although it was emphasized that survey responses were anonymous, there may remain some degree of social desirability bias or underrepresentation of more critical perspectives.

Comparisons with other European countries with similar decentralized systems remains a challenge given the limited number of similar studies published internationally. It is hoped that this study will serve as a useful tool for other countries wishing to carry out future assessments examining the attributes of internal completeness, acceptability, and usefulness.

### Recommendations and Conclusions

Surveillance of TB is inherently challenging given the typically long-term course of the disease and treatment within the context of complex socioeconomic risk factors. This study indicated that completeness of TB surveillance data in Spain has improved following the introduction of annual feedback reports in 2022, although variation persists by variable and region. Importantly, the combination of quantitative and qualitative analyses highlights not only the measurable progress in surveillance quality but also the perceptions, barriers, and enablers that shape the system’s performance. Although the reasons underlying observed improvements can be uncertain and are likely multifaceted, feedback reports were valued by regional stakeholders and appear to support a culture of continuous quality improvement.

Our findings suggest that the CNE should continue to send annual feedback reports to regions; however, they could be refined to be of even more benefit, for example, by focusing on a smaller number of key variables and indicators that align with those set out by the WHO guidance on TB surveillance published in 2024 [23]. This guidance defines a set of core indicators for countries with a case-based digital surveillance system as well as recommended reporting intervals, visualization, and interpretation for these indicators. This would support regions to target efforts where they are most effective and facilitate comparison with other countries. More broadly, strengthening alignment with international standards may help Spain position itself within global TB monitoring frameworks and contribute more effectively to regional benchmarking efforts.

In addition, the CNE should provide improved guidance on follow up of individuals who move between CCAAs or internationally during the long treatment course and how information from these individuals should be reported in the surveillance system. This could be supported by encouraging use of the ECDC “International tuberculosis care transfer form” [24]. This tool aims to support cross-border collaboration between health care providers to ensure the care needs for people with TB are transferred securely and efficiently, minimizing delays and reducing the risk of treatment interruption. In addition, the availability of a national personal identifier in Spain would facilitate interoperability of laboratory and surveillance systems within regions, as well as support easier transfer of care of patients between regions. These improvements would not only enhance data quality but also strengthen continuity of care, an essential component of TB control in decentralized health systems.

To fully realize the potential of TB surveillance, future assessments could consider expanding beyond completeness and perceptions of feedback reports, for example using standardized methods to carry out a formal evaluation and examining a more comprehensive list of attributes, such as validity, timeliness, representativeness, and sensitivity, in line with established frameworks [11,21,25]. Future evaluations could be also carried out using the WHO TB surveillance guidelines [23] as a framework, with completeness of data fields measured against those required to calculate the recommended indicators. In addition, to examine the possible determinants of changes observed beyond descriptive analysis, approaches such as multilevel logistic regression analysis with adjustment for covariates including CCAA could be considered to complement future evaluations. Finally, embedding assessments into a continuous improvement cycle, as already initiated by revising the most recent feedback reports, will help ensure that the system evolves and continues to provide reliable and actionable information that meet both national and international TB control objectives.

## Supplementary material

10.2196/93719 Multimedia Appendix 1 Example feedback report, example letter, and survey.

10.2196/93719 Multimedia Appendix 2 Mean percentage completeness and percentage of internal completeness.

## Abbreviations

AI: artificial intelligence
CCAA: autonomous community of Spain
CDC: Centers for Disease Control and Prevention
CNE: National Epidemiology Center (Centro Nacional de Epidemiología in Spanish)
ECDC: European Center for Disease Prevention and Control
EEA: European Economic Area
LOPDGDD: Ley Orgánica 3/2018 de Protección de Datos Personales y Garantía de los Derechos Digitales
RENAVE: The National Epidemiological Surveillance Network (La Red Española de Vigilancia Epidemiológica in Spanish)
TB: tuberculosis
WHO: World Health Organization
