# Supplementary material for: Data Completeness and Acceptability of Annual Feedback Reports and Tuberculosis Surveillance in Spain, 2018-2024: Mixed Methods Analysis
Source: JMIR Public Health Surveill. 2026 Sep 16;12:e93719. doi: 10.2196/93719 (PMC13580891; doi:10.2196/93719)
Supplement: Supplementary file 1 [file publichealth-v12-e93719-s001.docx]

# **Supplementary Material 1: Example feedback report circulated to one autonomous community in 2024, applying to 2023 annual data submission.**

**Original Spanish**

Buenos días,

Desde el Departamento de vigilancia de enfermedades transmisibles del CNE-ISCIII, como responsables de la gestión del sistema de vigilancia de la RENAVE, queremos mejorar la calidad de la información de algunas de las enfermedades sometidas a planes de vigilancia o control o relevantes para la vigilancia por otros motivos. Para ello hemos diseñado unas salidas y tablas que resumen la calidad de la información cargada en SiViEs de dichas enfermedades. Os enviamos la referente a la carga de tuberculosis de vuestra **CA [redacted]** hecha con fecha **19-06-2024 para los datos cargados de 2023**.

**Dado que sólo tenemos una información parcial de la actividad de vigilancia y seguimiento de cada caso, algunos de los comentarios que se incluyen a continuación podrían no aplicar y deben ser ignorados.**

Información que debería ser revisada:

- No hay datos para las siguientes variables:
  - Provincia y municipio donde se produjo el caso (sólo registrado en 1 caso). ¿Tenéis esta información en las encuestas, pero hay algún problema en el volcado o con el transformador? ¿O es asumible que la información puede ser imputada por lugar de residencia?
  - Fecha de diagnóstico (FDiag): ¿Tenéis esta información en las encuestas, pero hay algún problema en el volcado o con el transformador?
- Falta información sobre:
  - Edad en meses: Hay 1 caso de 1 año que da error por estar registrado como 12 meses
  - Importado: sólo hay un caso en el que no figura el país de origen.
  - Agente causal: Hay 379 casos sin agente casual y clasificación, de los cuales 19 son confirmados y 161 probables. Revisar especialmente estos últimos, por si tuvieran alguna prueba diagnóstica que pudiera hacerlos asumibles a “Mycobacterium tuberculosis, complejo”.
  - Clasificación: Hay 9 casos en los que no figura la clasificación pero se han asumido como confirmados, probables o sospechosos según los resultados de laboratorio, por si queréis contrastarlos.
  - Localización: Información relevante. Falta en 13 casos
  - Defunción: hay 512 registros sin información en 2023 y 56 en 2022. ¿Podría extrapolarse del resultado del tratamiento?
  - Resultado del tratamiento: falta en 496 casos de 2023 y en 25 casos de 2022 (¿serían perdidas en este ultimo caso? Revisar y actualizar cuando sea posible.
  - Resistencias y resultados de antibiogramas. Información muy relevante. Hay 396 casos en los que no figura si se realizó estudio de resistencias. De hecho, sólo hay valores “si” registrados para esta variable. ¿Se podría asumir que en el resto (los 396 casos comentados) no se hizo estudio de resistencias?.

El resto de información cargada en SiViEs está mayoritariamente dentro del estándar de calidad, no obstante, se recomienda revisar la tasa de cumplimentación de cada una de las variables ya que aún existe posibilidad de mejora (hoja “Tasa_Cumplimentación”).

En el párrafo anterior hemos señalado la información que debería mejorarse y valorar si se puede actualizar la información en SiViEs. Para evitar duplicados en SiViEs, la opción más adecuada es reemplazar toda la base de datos con la opción de SiViEs**:** Añadir/actualizar con reemplazo toda la base. En cualquier caso, ante cualquier duda podéis consultar a [redacted].

**English translation**

Good morning,

From the Department of Surveillance of Communicable Diseases at the CNE‑ISCIII, as those responsible for managing the RENAVE surveillance system, we aim to improve the quality of the information for certain diseases that are subject to surveillance or control plans, or that are relevant for surveillance for other reasons. To this end, we have designed outputs and tables that summarise the quality of the information uploaded to SiViEs for these diseases. We are sending you the one corresponding to the tuberculosis data uploaded by your Autonomous Community [redacted], generated on 19‑06‑2024 for the data uploaded for 2023.

**Since we only have partial information on the surveillance and follow‑up activity for each case, some of the comments included below may not apply and should be disregarded.**

Information that should be reviewed:

- There are no data for the following variables:
  - *Province and municipality where the case occurred* (only recorded for 1 case). Do you have this information in the questionnaires, but there is a problem with the upload or with the transformer? Or is it acceptable to impute this information based on place of residence?
  - *Date of diagnosis (FDiag)*: Do you have this information in the questionnaires, but there is a problem with the upload or with the transformer?
- Missing information on:
  - *Age in months*: There is 1 case of a 1‑year‑old that shows an error because it is recorded as 12 months.
  - *Imported*: There is only one case for which the country of origin is not recorded.
  - *Causal agent*: There are 379 cases without causal agent and classification, of which 19 are confirmed and 161 probable. Please review these latter cases in particular, in case they have any diagnostic test that would allow them to be classified as “Mycobacterium tuberculosis complex”.
  - *Classification*: There are 9 cases without classification, but they have been assumed to be confirmed, probable, or suspected based on laboratory results, in case you wish to verify them.
  - *Site of disease*: Relevant information. Missing in 13 cases.
  - *Death*: There are 512 records without information in 2023 and 56 in 2022. Could this be extrapolated from the treatment outcome?
  - *Treatment outcome*: Missing in 496 cases from 2023 and in 25 cases from 2022 (could these be losses to follow‑up in the latter year?). Please review and update when possible.
  - *Drug resistance and antibiogram results*: Very relevant information. There are 396 cases for which it is not recorded whether a resistance study was performed. In fact, only “yes” values are recorded for this variable. Could it be assumed that in the remaining 396 cases no resistance study was performed?

The rest of the information uploaded to SiViEs is mostly within the quality standard; however, it is recommended to review the completion rate of each variable, as there is still room for improvement (see “Tasa_Cumplimentación” sheet).

In the paragraph above, we have highlighted the information that should be improved and assessed to determine whether it can be updated in SiViEs. To avoid duplicates in SiViEs, the most appropriate option is to replace the entire database using the SiViEs option: *Add/update with full replacement of the database*. In any case, if you have any questions, you may contact [redacted].

# **Supplementary Material 2: Example letter accompanying quantitative analysis sent to each CCAA alongside the qualitative survey to be completed**

**Original Spanish**

Estimad@ colega,

Nos gustaría invitarle a participar en una breve encuesta para recopilar información sobre el informe de evaluación anual que enviamos cada año para mejorar la calidad de los datos de vigilancia de la tuberculosis (TB).

Desde 2021, el Centro Nacional de Epidemiología (CNE) del Instituto de Salud Carlos III (ISCIII) envía a cada CCAA informes anuales personalizados de evaluación sobre la exhaustividad de las variables clave notificadas a SIVIES el año anterior. El objetivo de estos informes es ayudar a las CCAA a identificar áreas de mejora en la vigilancia para el año siguiente y mejorar la calidad de la información recopilada a nivel nacional. Mejorar esta información es clave para tener una visión más completa y comprehensiva de la TB y contribuir a la mejora de su prevención y control.

Hemos realizado un análisis comparando los periodos anteriores y posteriores a la implementación de estos informes (2018-2020 y 2021-2023), y encontramos que, globalmente, la exhaustividad ha mejorado en un 10,2%, pasando de un 66,0% en el primer periodo a un 76,2% en el segundo periodo (Figura 1). Esta mejora se distribuye de la siguiente manera: un 2,5% las variables relacionadas con el paciente, un 12,8% las variables relacionadas con la enfermedad y un 13,3% las variables relacionadas con las pruebas de laboratorio (Figuras 2-4). Las variables analizadas se encuentran en el Anexo 1.

En su CCAA, la exhaustividad ha cambiado en un 9,2% (76,4% primer periodo, 85,6% segundo periodo), con un 3% para las variables del paciente, un 9,3% para las variables relacionadas con la enfermedad y un 16,8% para las variables de laboratorio (ver Figuras 1-4).

**Nos gustaría conocer su opinión sobre los informes anuales de evaluación, su utilidad, posibles barreras para la notificación, así como sugerencias para mejorar la notificación de la TB.** Por ello, le agradeceríamos si pudiera completar la siguiente encuesta de 12 preguntas, cuya duración estimada es de 10 minutos, antes del **12 de mayo de 2025.**

Las respuestas serán analizadas de manera conjunta para todas las CCAA y no se compartirán respuestas individuales, manteniéndose el anonimato de los/as participantes. Además, vuestras aportaciones serán tenidas en cuenta en la evaluación de los datos de 2024. Con los resultados agregados se elaborará un informe que se difundirá entre las CCAA y, si es pertinente y con el consentimiento de las CCAA participantes, los resultados podrían enviarse a una revista indexada para su publicación. En caso de que se desarrolle un artículo, se invitará a las CCAA a formar parte de un grupo de trabajo y serán incluidas como autoras.

Muchas gracias por su tiempo y colaboración.

**English translation**

Dear colleague,

We would like to invite you to participate in a brief survey to gather information about the annual evaluation report that we send each year to improve the quality of tuberculosis (TB) surveillance data.

Since 2021, the National Centre for Epidemiology (CNE) of the Instituto de Salud Carlos III (ISCIII) has been sending each Autonomous Community (CCAA) personalised annual evaluation reports on the completeness of key variables reported to SIVIES during the previous year. The aim of these reports is to help the CCAA identify areas for improvement in surveillance for the following year and to enhance the quality of the information collected at the national level. Improving this information is essential to obtain a more complete and comprehensive picture of TB and to contribute to better prevention and control.

We have conducted an analysis comparing the periods before and after the implementation of these reports (2018–2020 and 2021–2023), and we found that, overall, completeness has improved by 10.2%, increasing from 66.0% in the first period to 76.2% in the second period (Figure 1). This improvement is distributed as follows: a 2.5% increase in patient‑related variables, a 12.8% increase in disease‑related variables, and a 13.3% increase in laboratory‑related variables (Figures 2–4). The variables analysed are listed in Annex 1.

In your CCAA, completeness has changed by 9.2% (76.4% in the first period, 85.6% in the second period), with a 3% increase in patient variables, a 9.3% increase in disease‑related variables, and a 16.8% increase in laboratory variables (see Figures 1–4).

We would like to know your opinion about the annual evaluation reports, their usefulness, possible barriers to reporting, and suggestions for improving TB notification. Therefore, we would be grateful if you could complete the following 12‑question survey, which is estimated to take 10 minutes, before 12 May 2025.

Responses will be analysed collectively for all CCAA, and individual responses will not be shared, ensuring participant anonymity. In addition, your contributions will be taken into account in the evaluation of the 2024 data. An aggregated report will be prepared and shared with the CCAA, and, if appropriate and with the consent of the participating CCAA, the results may be submitted to a peer‑reviewed journal for publication. If an article is developed, the CCAA will be invited to join a working group and will be included as authors.

Thank you very much for your time and collaboration.

# **Supplementary Material 3: survey circulated to regions**

# **Original Spanish**

1. **¿Cree que es importante tener datos de vigilancia de la tuberculosis (TB) de alta calidad a nivel nacional?**

Muy importante; importante; neutral; poco importante; nada importante

2. **¿Está satisfecho/a con el sistema nacional actual de vigilancia y notificación de datos de TB en España?**

Muy satisfecho; satisfecho; neutral; insatisfecho; muy insatisfecho

3. **¿Está satisfecho/a con el nivel de exhaustividad de los datos de vigilancia de TB que reporta su CCAA al sistema nacional?**

Muy satisfecho; satisfecho; neutral; insatisfecho; muy insatisfecho

4. **¿Cómo de útiles considera los informes anuales de evaluación que le envía el CNE para mejorar la exhaustividad de los datos de vigilancia de TB en su CCAA?**

Extremadamente útiles; muy útiles; moderadamente útiles; poco útiles; nada útiles

5. **Si cree que los informes anuales de evaluación han sido útiles, ¿puede explicar cómo o en qué aspectos?**
6. **Si no considera que los informes anuales de evaluación hayan sido útiles, ¿puede explicar por qué no? ¿Hay algo que los haría más útiles?**
7. **¿Considera que el formato de estos informes es adecuado?**

Sí; parcialmente; no

8. **Si considera que el formato no es adecuado, o es parcialmente adecuado, por favor explica cómo se podría los mejorar los informes en este aspecto**
9. **¿Considera que el contenido de estos informes es adecuado?**

Sí; parcialmente; no

10. **Si considera que el contenido no es adecuado, o es parcialmente adecuado, por favor explica cómo se podría los mejorar los informes en este aspecto**
11. **¿Cree que los informes anuales de evaluación se distribuyen en un momento que permite implementar mejoras antes de la siguiente entrega anual de datos?**

Sí, en el momento es adecuado; No, se envían demasiado tarde; No, se envían demasiado pronto

12. **¿Se difunde la información de los informes anuales de calidad elaborados por el CNE a otros colegas dentro de su CCAA?**

Sí; no

13. **Si difunde la información a otros colegas, ¿a quién y cómo?**
14. **¿Cuáles son las principales barreras que enfrenta su CCAA para lograr una notificación más completa de las variables clave de TB?**
15. **¿Existe actualmente alguna medida implementada en su CCAA para apoyar la mejora de la exhaustividad en la notificación de datos de vigilancia de TB?**

Sí; no

16. **Si existe alguna medida, ¿podría decirnos cuáles son?**
17. **Por favor, agregue cualquier comentario adicional que desee compartir, por ejemplo, sugerencias sobre cómo mejorar los informes anuales de evaluación o como se podría mejorar la notificación de la vigilancia de TB a nivel de su CCAA y a nivel nacional**

**English translation**

1. **Do you think it is important to have high‑quality national‑level tuberculosis (TB) surveillance data?**

Very important; important; neutral; slightly important; not important

2. **Are you satisfied with the current national system for TB surveillance and data reporting in Spain?**

Very satisfied; satisfied; neutral; dissatisfied; very dissatisfied

3. **Are you satisfied with the level of completeness of the TB surveillance data that your Autonomous Community (CCAA) reports to the national system?**

Very satisfied; satisfied; neutral, dissatisfied; very dissatisfied

4. **How useful do you find the annual evaluation reports sent by the CNE for improving the completeness of TB surveillance data in your CCAA?**

Extremely useful; very useful; moderately useful; slightly useful; not useful

5. **If you believe the annual evaluation reports have been useful, can you explain how or in what ways?**
6. **If you do not consider the annual evaluation reports useful, can you explain why not? Is there anything that would make them more useful?**
7. **Do you consider the format of these reports appropriate?**

Yes; partially; no

8. **If you consider the format inappropriate or only partially appropriate, please explain how the reports could be improved in this regard.**
9. **Do you consider the content of these reports appropriate?**

Yes; partially; no

10. **If you consider the content inappropriate or only partially appropriate, please explain how the reports could be improved in this regard.**
11. **Do you think the annual evaluation reports are distributed at a time that allows improvements to be implemented before the next annual data submission?**

Yes, the timing is appropriate; No, they are sent too late; No, they are sent too early

12. **Is the information from the annual quality reports prepared by the CNE shared with other colleagues within your CCAA?**

Yes; no

13. **If you share the information with other colleagues, with whom and how?**
14. **What are the main barriers your CCAA faces in achieving more complete reporting of key TB variables?**
15. **Is there currently any measure implemented in your CCAA to support improved completeness in TB surveillance data reporting?**

Yes; no

16. **If such measures exist, could you tell us what they are?**
17. **Please add any additional comments you would like to share, for example, suggestions on how to improve the annual evaluation reports or how TB surveillance reporting could be improved at the CCAA level and at the national level.**
