# Supplementary material for: Data Completeness and Acceptability of Annual Feedback Reports and Tuberculosis Surveillance in Spain, 2018-2024: Mixed Methods Analysis
Source: JMIR Public Health Surveill. 2026 Sep 16;12:e93719. doi: 10.2196/93719 (PMC13580891; doi:10.2196/93719)
Supplement: Supplementary file 2 [file publichealth-v12-e93719-s002.docx]

# **Table S1. Mean percentage completeness of 40 TB variables reported by autonomous community (CCAA) in period 1 (2018-2020) and period 2 (2021-2023), difference between periods, and paired t-test p-value for the difference, Spain.**

| **Autonomous community** | **Period 1 mean completeness** | **Period 2 mean completeness** | **Difference between means** | **p-value for paired test** |
| --- | --- | --- | --- | --- |
| Andalusia | 55.3 | 66.5 | +11.2 | <.001 |
| Aragon | 47.5 | 72.6 | +25.1 | <.001 |
| Asturias | 88.7 | 83.5 | -5.2 | <.001 |
| The Balearic Islands | 73.3 | 73.8 | +0.5 | 0.22 |
| The Canary Islands | 85.3 | 86.6 | +1.3 | 0.09 |
| Cantabria | 76.6 | 80.6 | +4.0 | 0.21 |
| Castilla y Leon | 57.8 | 74.9 | +17.2 | 0.001 |
| Castilla la Mancha | 57.4 | 56.9 | -0.5 | 0.67 |
| Catalonia | 74.7 | 83.3 | +8.6 | 0.006 |
| Valencia | 55.1 | 81.3 | +26.2 | <.001 |
| Extremadura | 73.0 | 78.2 | +5.2 | <.001 |
| Galicia | 88.4 | 87.5 | -0.9 | 0.01 |
| Madrid | 44.9 | 62.6 | +17.6 | 0.005 |
| Murcia | 76.4 | 85.3 | +8.9 | 0.01 |
| Navarra | 59.5 | 71.6 | +12.2 | 0.10 |
| The Basque Country | 67.3 | 77.9 | +10.6 | 0.003 |
| Rioja Rio | 84.0 | 87.5 | +3.5 | 0.48 |
| Ceuta | 60.1 | 77.9 | +17.8 | <.001 |
| Melilla | 72.9 | 79.0 | +6.1 | 0.008* |

*p-value for the Shapiro-Wilks test indicated normality of distribution (p=0.17) therefore paired t-test was used. All other p-values are for Wilcoxon signed-rank test as differences were found to be non-normally distributed.

# **Table S2: Percentage internal completeness of 40 variables for 17,534 reported TB cases excluding the COVID-19 pandemic years (period 1 (2018-2019) and period 2 (2022-2023)) by variable group: means, medians, and statistical test results**

| **Variable group** | **Mean completeness period 1 (%)** | **Mean completeness period 2 (%)** | **Difference (period 2 – period 1)** | **Shapiro-Wilk**  **p-value (normality)** | **Paired difference test**  **p-value** |
| --- | --- | --- | --- | --- | --- |
| Patient variables | 80.0 | 82.4 | 2.4 | .01 | 1 |
| Illness variables | 64.2 | 79.0 | 14.8 | .10 | <.001* |
| Laboratory variables | 52.1 | 71.0 | 18.9 | .06 | <.001* |
| All variables | 65.2 | 77.7 | 12.5 | .002 | <.001 |

*p-value for the Shapiro-Wilks test indicated normality of distribution of the differences (p>.05) therefore paired t-test was used. All other p-values are for Wilcoxon signed-rank test as differences were found to be non-normally distributed.
